# A National Assessment of the Epidemiology of Severe Fever with Thrombocytopenia Syndrome, China

**DOI:** 10.1038/srep09679

**Published:** 2015-04-23

**Authors:** Kun Liu, Hang Zhou, Ruo-Xi Sun, Hong-Wu Yao, Yu Li, Li-Ping Wang, Di Mu, Xin-Lou Li, Yang Yang, Gregory C. Gray, Ning Cui, Wen-Wu Yin, Li-Qun Fang, Hong-Jie Yu, Wu-Chun Cao

**Affiliations:** 1 The State Key Laboratory of Pathogen and Biosecurity, Beijing Institute of Microbiology and Epidemiology, Beijing 100071, P. R. China; 2 Division of Infectious Disease, Key Laboratory of Surveillance and Early-warning on Infectious Disease, Chinese Centre for Disease Control and Prevention, Beijing 102206, P. R. China; 3 Anhui Medical University, Hefei, 230032, P. R. China; 4 Department of Biostatistics, College of Public Health and Health Professions, and Emerging Pathogens Institute, University of Florida, 32311, Florida, USA; 5 Duke University School of Medicine, Durham, 27710, North Carolina, USA; 6 The 154 Hospital, People's Liberation Army, Xinyang, 464000, P.R. China

## Abstract

First discovered in rural areas of middle-eastern China in 2009, severe fever with thrombocytopenia syndrome (SFTS) is an emerging tick-borne zoonosis affecting hundreds of cases reported in China each year. Using the national surveillance data from 2010 to 2013, we conducted this retrospective epidemiological study and risk assessment of SFTS in China. We found that the incidence of SFTS and its epidemic areas are continuing to grow, but the case fatality rate (CFR) has steadily decreased. SFTS most commonly affected elderly farmers who acquired infection between May and July in middle-eastern China. However, other epidemiological characteristics such as incidence, sex ratio, CFR, and seasonality differ substantially across the affected provinces, which seem to be consistent with local agricultural activities and the seasonal abundance of ticks. Spatial scan statistics detected three hot spots of SFTS that accounted for 69.1% of SFTS cases in China. There was a strong association of SFTS incidence with temporal changes in the climate within the clusters. Multivariate modeling identified climate conditions, elevation, forest coverage, cattle density, and the presence of *Haemaphysalis longicornis* ticks as independent risk factors in the distribution of SFTS, based on which a predicted risk map of the disease was derived.

In 2009, severe fever with thrombocytopenia syndrome (SFTS) was first discovered in rural areas of middle-eastern China. As an emerging zoonotic infectious disease, SFTS is recognized to cause hundreds of symptomatic human infections each year. The causative agent is a newly recognized bunyavirus named SFTS virus (SFTSV) of the *Phlebovirus* genus. The disease normally manifests itself with fever, thrombocytopenia, leukocytopenia, and gastrointestinal symptoms. SFTS has an average case-fatality rate of 12% but could be as high as 30% for some populations^1^,^2^,^3^. SFTSV has been widely detected and isolated from two tick species (*Haemaphysalis longicornis* and *Rhipicephalus microplus*) in epidemic areas, suggesting that these ticks are the most likely vector for transmitting the virus to humans^4^. Domestic animals are thought to be the reservoirs for the virus, as SFTSV antibodies have been found in goats, cattle, sheep, pigs and dogs^5^,^6^. Humans may also be infected through contact with infected patient's blood^7^,^8^. According to the surveillance data managed by the Chinese Center for Disease Control and Prevention, as of December 2013, SFTS cases had been reported in 14 provinces of China, and epidemic areas are seemingly expanding. Furthermore, SFTS has also been recognized in Japan and South Korea in 2012, and a disease similar to SFTS has been reported in the United States^9^,^10^,^11^. Hence, SFTSV or similar viruses have a wider distribution and may be more important than was previously thought, and raise an increasingly important threat to the global health.

As a novel tick-borne disease, SFTS chiefly occurs among farmers who reside in wooded and hilly areas^12^,^13^,^14^. Some patients who later are diagnosed with SFTS, report being bitten by ticks before the onset of their symptoms^3^,^15^. The disease usually presents during March to November, peaking between May and July, and is mainly distributed in the rural areas of Hubei, Henan, Shandong, and Anhui provinces^2^,^13^. Exposure to ticks, living and working in weeds and shrubs were found to be important risk factors in previous analyses^14^,^15^,^16^. However, those results were based on a small number of patients living in only a few regions of China.

In 2010, the Chinese Ministry of Health initiated a national SFTS surveillance program, requiring national reporting. In this report we examined these new data which were archived in the China Information System for Diseases Control and Prevention (CISDCP)^17^. We conducted this retrospective study with three major objectives: (1) to provide an comprehensive epidemiological overview of human SFTS in China; (2) to investigate spatial, temporal, climate, and ecological risk factors for human SFTS cases; and (3) to map the potential risk distribution of SFTS in China.

## Results

### Prevalence Overview

**Figure 1:**
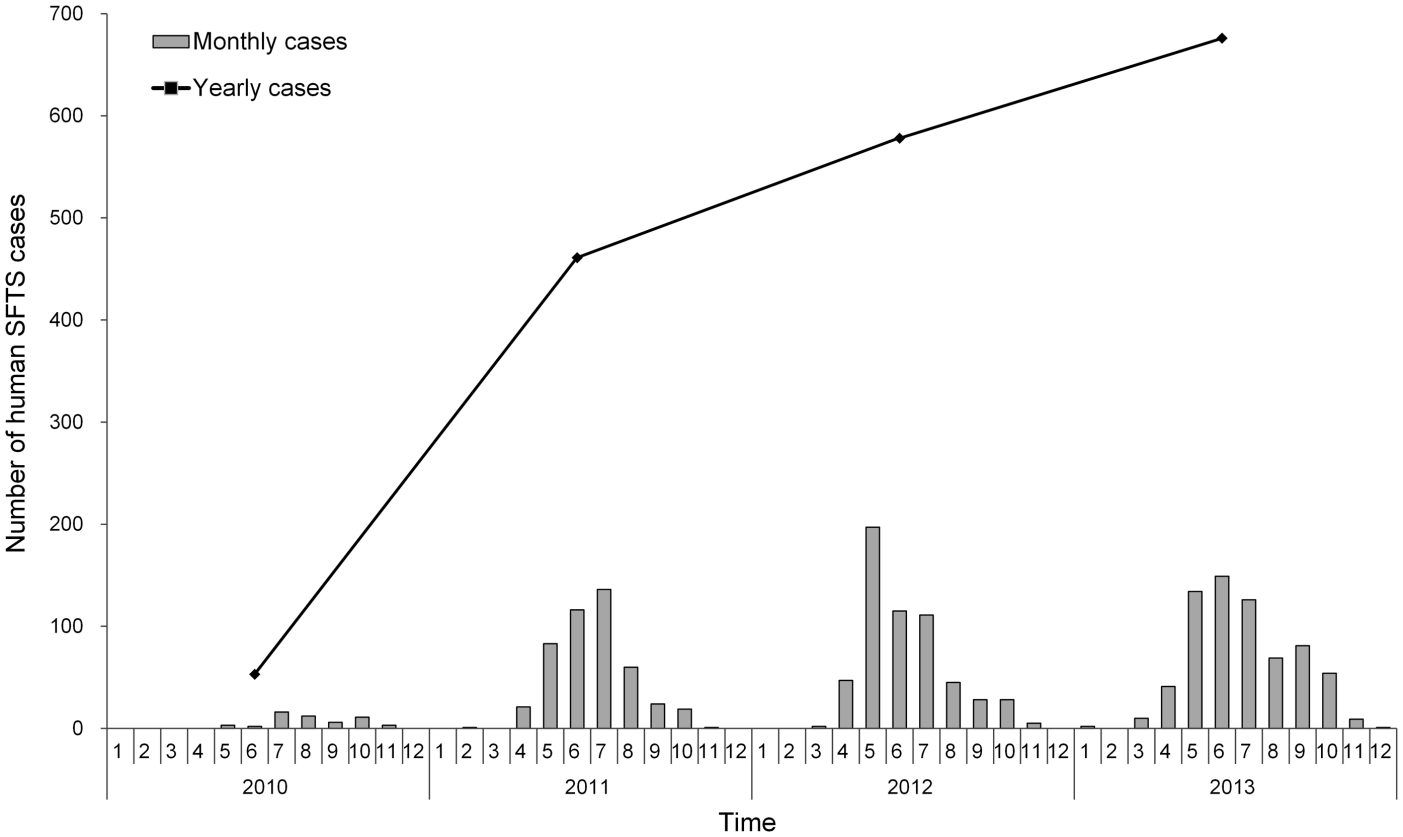
Temporal distribution of the human SFTS cases in China from 2010 to 2013. The histogram represents the monthly number of SFTS cases, and the line represents the annual number of SFTS cases.

**Figure 2:**
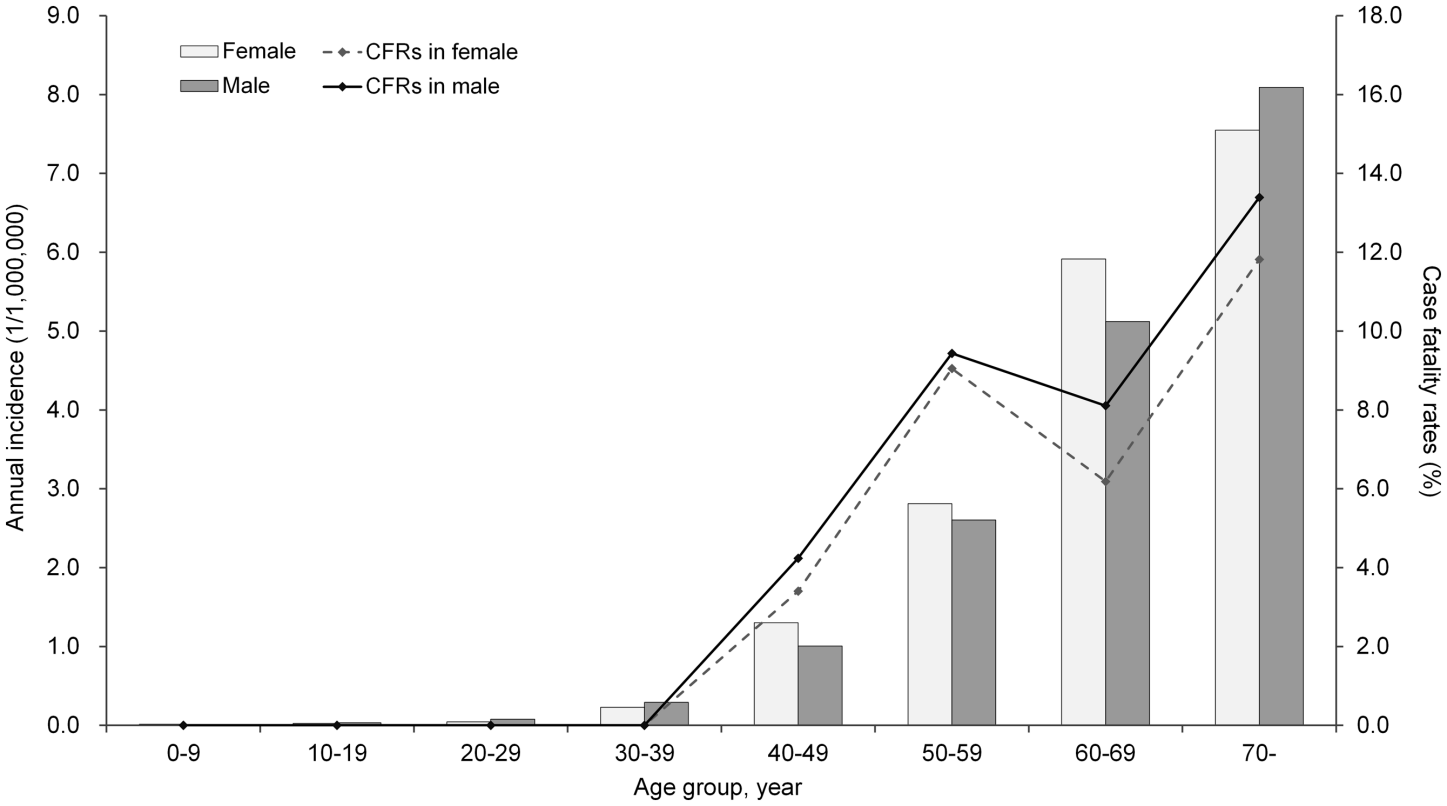
Age and sex distribution of the SFTS incidence and CFRs in China.

Between 2010 and 2013, a total of 1,768 laboratory-confirmed SFTS cases were reported in China. During this 4 years period, the annual number of SFTS cases steadily increased from 53 cases (in 2010) to 676 cases (in 2013) per year. The national SFTS epidemic curve revealed significant seasonality, with 67.2% cases occurring between May and July (Figure 1). The median age of the patients was 61 years (range, 1–93), and the majority of cases were 50 years of age or older (1,494, 84.5%). The incidences increased from younger to older age groups (χ^2^ test for trend, P = 0.017, 0.020, respectively), but no sex difference was observed with a female-to-male ratio of 1.06:1 (Figure 2). Most cases were farmers or forest workers (94.4%), who lived in rural areas and engaged in the agricultural activities.

Of the 1,768 confirmed SFTS cases, 145 deaths were confirmed to be associated with SFTS, with an overall fatality rate of 8.2%. Annual case fatality rates exhibited a downward trend from 15.1% to 7.1% from 2010 to 2013 (Table 1). The median age of the deaths was 66 years (range, 40–86), and 76 (52.4%) of deaths occurred in males. Case fatality rates in males and females both increased with age (χ^2^ test for trend, P = 0.018, 0.015, respectively), but no difference was found between genders (Figure 2).

### Geographic differences

A comparative analysis of the surveillance data in different epidemic provinces showed significant differences not only in disease incidence but also with respect to demographics, CFRs, and seasonality.

By the end of 2013, SFTS cases had been reported in 14 provinces of China. Henan Province reported the highest incidence of SFTS, followed by Shandong Province, Hubei Province, Liaoning Province, Anhui Province, and Zhejiang Province, the incidences being 0.73, 0.53, 0.38, 0.27, 0.20, and 0.12 per 100,000 persons, respectively. The other seven provinces reported only sporadic SFTS cases. Among the affected provinces, the sex ratio in SFTS cases was dramatically different between Henan Province and all other provinces, with a female-to-male ratio of 1.62 (425/263) in the former, and 0.82 (485/595) in all other provinces combined (p-value < 0.001). Liaoning Province had the lowest ratio of 0.71 (50/70). Also in Henan Province, the CFR was 2.8%, significantly lower than other provinces which ranged from 8.7 to 12.3% (Table 1 and Figure S1). In addition, the SFTS epidemic peaked later in provinces with higher latitudes. From south to north, the peak months were May to July in Henan, Anhui and Hubei provinces combined (most cases appeared in the junction region of the three provinces), between June and July in Shandong Province, and between July and August in Liaoning Province (Figure S2).

### Spatial-temporal clusters

**Figure 3:**
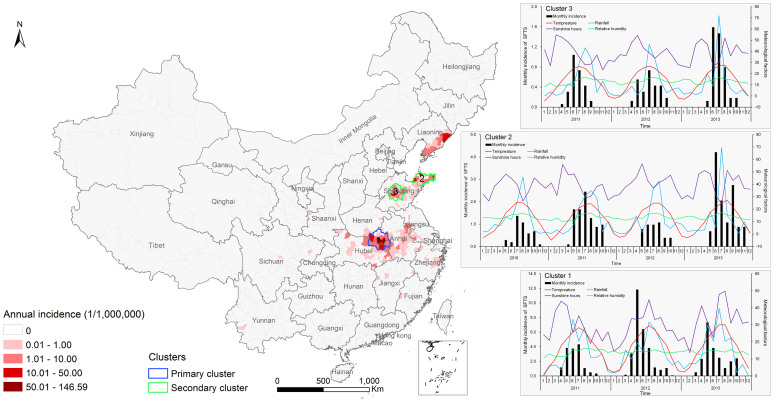
Spatial-temporal clusters overlapping the annual incidence of SFTS in China, and the relationships between monthly SFTS incidence and climate factors in each cluster. In order to show the relationships in the same scale, the values of monthly cumulative rainfall, monthly cumulative sunshine hours, and monthly average relative humidity were divided by five, respectively. The map was created in ArcGIS 9.2 software (ESRI Inc., Redlands, CA, USA).

The spatial-temporal cluster analysis identified three clusters encompassing 59 counties mainly in the middle-eastern China (Figure 3), where 2.9% of the national total population accounted for 69.1% of SFTS cases in China (Table 2). The primary cluster (cluster 1) was located at the junction of Henan, Hubei and Anhui provinces including 17 counties and a population of nearly 12 million. This cluster had a 152.9 relative risk (RR) value (p < 0.001), and spanned from April 2011 to October 2013. Cluster 2 was located in 18 counties of Jiaodong peninsula in Shandong Province with a RR of 30.0 (p < 0.001), and emerged between May 2010 and November 2013. Cluster 3 was located in the central of Shandong Province. It included 24 counties with a RR of 12.4 (p < 0.001), occurring between May 2011 and November 2013.

Spearman correlation analyses within the three clusters showed that climate factors were significantly associated with the temporal variation in SFTS incidence. Temperature was found to have the maximum correlation with the monthly incidence of SFTS, followed by rainfall, sunshine hours, and relative humidity (Table S2). Relative humidity was not correlated with SFTS incidence in cluster 1. Figure 3 shows the temporal variation in climate factors and the monthly SFTS incidence in the clusters.

### Risk Assessment

Based on the BRT model, the occurrence of SFTSV human infection was found to be significantly associated with eight predictors: temperature, rainfall, relative humidity, sunshine hours, elevation, distribution of *H. longicornis* ticks, cattle density, and coverage of forest (Table 3), all with average BRT weights >5.0%. The model-fitted risks were plotted versus each predictor in Figure S3, showing non-linear relationships between the predictors and the risk of SFTS. The predicted risk of SFTS occurrence first increased significantly and then plateaued in response to increase in rainfall, sunshine hours, cattle density or percentage coverage of forest. With increasing temperature and relative humidity, the predicted risk also increased first, but dropped dramatically passing the peak. A strong negative relationship was found for elevation, with the highest risk corresponding to low elevations of 100 ~ 400 m, and then a declining trend for higher elevations. In addition, a relative higher risk was found in areas where *H. longicornis* ticks had been previously identified.

**Figure 4:**
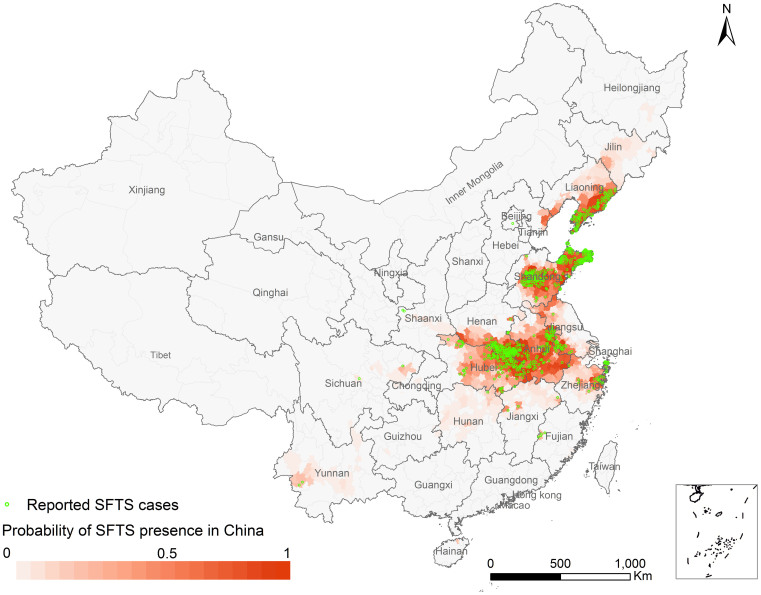
The predicted risk distribution of SFTS at the county level in China. The different color grades represent the predicted risk of occurrence of human SFTSV infection, and green triangles represent the observed SFTS cases from 2010 to 2013. The map was created in ArcGIS 9.2 software (ESRI Inc., Redlands, CA, USA).

The estimated AUC value of 0.985 (95% CI 0.975–0.994) indicated an excellent predictive accuracy of the model. As expected, the AUC estimated by the training data (0.993, 95% CI 0.983–0.998) was better than that estimated using the test dataset (0.968, 95% CI 0.953–0.982) (Figure S4). On the basis of the average predicted probabilities, a risk map for SFTSV human infections in China was created at the county level (Figure 4). The high risk areas cover a wide range of middle-eastern China, overlapping very well with the observed SFTS cases. Furthermore, some areas in Jilin, Liaoning, Anhui and Zhejiang provinces may also see SFTS cases emerging.

## Discussion

As an emerging infectious disease, SFTS is considered an increasingly important public health threat because of its wide distribution and high fatality rate^1^,^2^,^13^. In this study, we provided the first comprehensive epidemiological description and risk assessment of the human SFTSV infections in China in recent years. Nationally, the incidence of SFTS and the epidemic areas continued to grow over time. Most cases were elderly farmers who acquired infection between May and July in the rural areas of Henan, Hubei, Shandong, Anhui, and Liaoning provinces. However, epidemic features such as incidence, sex ratio, CFR, and seasonality showed marked differences across the affected provinces, probably due to the heterogeneity in the local agricultural activities and the distribution and lifecycle of ticks. Spatial scan statistics detected three significant spatiotemporal clusters of SFTS in China, and climate factors seemed to partially explain the temporal variation of SFTS. The BRT model identified climate conditions, elevation, forest coverage, *Haemaphysalis longicornis* ticks, and cattle density as important risk factors for human infection with SFTSV.

Since the emergence of the disease in 2009, ticks have been suggested to be the most likely vectors for SFTSV, and farmers performing agricultural activities in the fields or wooded areas are at the highest risk of infection^15^,^18^,^19^. In China, many young adults from poor rural areas migrate to cities for better job opportunities, leaving the elderly and children in the villages^20^. The elderly, in particular elderly males then have to assume the agricultural activities (including preparing land for cultivation, planting crops, pasturing cattle, and clearing weeds, etc.), exposing themselves to tick bites. As a result, more male cases than female cases were reported in the most places of China. Interestingly, more female than male cases were observed in Henan Province. Most human SFTS cases in Henan Province (98.8%, 680/688) came from Xinyang Prefecture, a typical tea-growing region (Figure S5). More elderly women than men are involved in tea-picking activities, especially when the vector tick (*H. longicornis*) is highly active in that region and imposing remarkably increased risk for SFTSV infection^3^,^14^,^18^. Nevertheless, as the most severely affected region in China, Xinyang Prefecture has implemented SFTS intervention programs including promoting public awareness, establishing sentinel hospitals, and improving clinicians' skills^13^,^14^,^21^,^22^. These actions likely resulted in more patients seeking medical care in hospitals and being diagnosed, which in turn led to more reported cases but a lower CFR in Henan Province as compared to other provinces (Figure S1). We also found that the timing of SFTS epidemic peaks seemed to be associated with latitude among the affected provinces, which seems likely to be related to the timing of seasonal abundance of ticks^3^,^13^,^14^,^19^,^20^,^21^,^22^,^23^,^24^.

In this study, three significant spatial-temporal “hotspots” areas of SFTS were detected in China using spatial scan statistics. These hotspots account for 69.1% of total SFTS cases with only 2.9% of the national total population. All the clustered areas and time frames share similar ecological environments characterized by a warm humid climate, mountainous or hilly-forested landscape, and a high abundance of *H. longicornis* ticks^14^,^18^,^19^,^20^,^21^,^22^,^23^. These areas should be prioritized for intervention efforts to control the disease.

The multivariate BRT modeling results confirmed the fundamental role of climate conditions and quantified their non-linear effects in SFTSV transmission. In general, the life cycle of ticks is very sensitive to the climate. Within a suitable range, higher temperatures shorten tick development periods, raise egg productivity and hatch-ratios, and promote biting behaviors^25^,^26^,^27^. Increasing rainfall and air humidity would lead to more breeding sites in luxuriant shrub or forest areas, and would hence increase the population size of the ticks^24^,^28^,^29^. Climate can also affect viral replication and transmission, host animal behaviors, and human outdoor exposures^27^,^28^,^29^. However, climate effects need not to be linear, for example, extreme temperatures can adversely affect the geographic distribution of ticks and suppress viral activity. Also excessive rainfall may flush out the tick breeding sites and destroy eggs and larvae^17^,^25^,^28^,^29^,^30^. While we have discovered the nonlinear effects of climate variables on the risk of human SFTSV infections, the mechanisms regarding how weather conditions change tick life cycles as well as how they impact human exposure needs to be further investigated.

In addition to climate variables, we found elevation, forest coverage and distribution of *H. longicornis* ticks to be useful predictors for human SFTSV infections. Most cases occurred in areas with low elevations ranging from 100 to 400 m, consistent with the height of hills or low-altitude mountains suitable for both ticks and human activities^3^,^14^. Forest is directly related to the SFTSV transmission via its impacts on breeding sites, tick survival, and human activities^24^,^28^,^31^. For example, the hilly-forested rural areas in Xinyang Prefecture are the most highly epidemic places in China^14^. Previous surveys have suggested that *H. longicornis* and *R. microplus* ticks are the most likely vectors in SFTSV transmission to humans^1^,^2^,^4^. However, our risk assessment model demonstrated that *H. longicornis* rather than *R. microplus* was more strongly associated with SFTSV human infections (Figure S6). We speculate that this might be related with natural habitats of the *R. microplus* ticks which heavily rely on cattle blood. Likewise, for domestic animals, previous studies have demonstrated goats and cattle were reservoirs for the virus, and goats were even more important for maintaining ticks^5^,^32^,^33^,^34^. Our analysis also identified a positive relationship between risk of human SFTS occurrence and cattle density, but the association between human SFTS and goat density was not significant. We speculate this phenomenon could be partially explained by the fact that cattle play an important role in the mountainous and hilly agriculture in mainland China. Compared to goats, cattle are more commonly raised for agriculture activities in many families, which could increase the human exposure of ticks though contacting with cattle. Certainly, the exact role of ticks and possible animal reservoirs for SFTSV should be addressed in better designed future studies.

Our risk assessment model had a good predictive power for identifying areas of SFTS in China. The predicted high risk areas overlapped well with the current reported SFTS cases, and some other areas in Jilin, Liaoning, Anhui and Zhejiang provinces might also emerge SFTS. In practical terms, the predicted map recommended public health officials to expand the surveillance program to all the high risk areas including the areas without cases reported now, and targeted interventions, such as disease and vector/host surveillance, seroprevalance investigation, diagnosis and healthcare service training should be enhanced. Meanwhile, people in epidemic areas should improve awareness of self-protection to avoid being bitten by ticks in outdoor activities, especially in spring and summer when tick is highly active^35^.

The study has some limitations worth mentioning. First, the hospital-based surveillance system only captures patients with SFTS who sought medical care, while patients with subclinical or mild infection are missed. Additionally, in some cases viral laboratory confirmation might not have always been available due to inadequate sample quality or laboratory limitations in rural areas. Hence, underreporting seems inevitable. Second, as an emerging infectious disease, the time period of the study was relative short, the increasing number of reported cases in recent years could be associated with the improvement of knowledge and diagnosis of SFTS, and it is believed that more cases will be detected in a wider scale of areas in the near future^20^. Third, due to data availability, some relevant individual characteristics of cases, such as immunological condition, socioeconomic status, exposure history, were not considered in the study. Finally, our ecological model assessed the relationship between the ecological factors and the occurrence of human SFTSV infections, but the complete transmission chain of SFTSV is likely more complicated. Other determinants such as the number of infected ticks, the abundance of key reservoir hosts, and the contact frequency between the infected ticks and humans were not included in our study due to the absence of such data.

In conclusion, our study shed lights on the epidemiological features, geographic differences, clustering patterns, ecological risk factors, and predicted risk map for human SFTSV infections. Further research work should focus on better understanding the likely more complex SFTSV ecologic transmission cycle and upon developing of an effective vaccine against SFTSV infection.

## Methods

### Data Collection and Management

Data regarding reported human cases of SFTS from January 1, 2010 to December 31, 2013 were collected from CISDCP. According to the national guidelines, a laboratory-confirmed SFTS case was defined as having at least one of the following criteria: 1) a positive SFTSV culture; 2) a positive result for SFTSV RNA by molecular detection; 3) seroconversion or ≥4-fold increase in specific antibody to SFTSV between acute and convalescent serum samples^17^. Information regarding age, sex, occupation, onset date of symptoms, and residential township were also collected for each case.

Data concerning climate, environmental and ecological factors were collected to study potential determinants for the spatiotemporal distribution of SFTS in China. Climate data including monthly averages temperature, relative humidity, and monthly accumulative rainfall, sunshine hours during the study period were obtained from Chinese Academy of Meteorological Sciences (www.cams.cma.gov.cn). Land cover data were derived from a raster version of the “GlobCover 2009 land cover map” with a resolution of 300 meter provided by the European Space Agency (available at http://ionia1.esrin.esa.int). Elevation data were obtained from the Shuttle Radar Topography Mission (SRTM) archives (http://www.srtm.csi.cigar.org). Demographic data regarding each county were obtained from the National Bureau of Statistics of China from the Sixth National Census in 2010. Raster-typed data with 5.5 km*5.5 km resolution regarding the density of goat and cattle were derived from the Food and Agriculture Organization of the United Nations (http://www.fao.org/AG/againfo/resources/en/glw/GLW_dens.html). Considering the important role of ticks in the SFTSV ecologic life cycle^1^,^4^, we collected the geographic distribution of two SFTS-related tick species (*H. longicornis* and *R. microplus*) in China (search strategy seeing in supplementary materials). Finally, a set of 13 potential predictors for the occurrence of human SFTSV infection were calculated for each county by using spatial analytic approaches in ArcGIS 9.2 software (ESRI Inc., Redlands, CA, USA) (Table S1).

### Estimates of epidemiology and geographic differences

To characterize the current epidemiological features of human SFTS cases in China, we assessed the demographic, temporal, and spatial patterns of the disease from 2010 to 2013. We created a monthly incidence bar chart, an annual incidence curve (all of China), and a spatial thematic map displaying the annual incidence at the county level. To examine geographic differences, we also performed a comparative analysis of SFTS in different epidemic provinces.

### Spatial-temporal clusters

To identify clusters of SFTS in China from 2010 to 2013, the spatial scan statistic was performed using SaTScan software (version 9.0, http://www.satscan.org). We structured a space-time discrete Poisson model to detect clusters, constraining the maximum temporal cluster size to be ≤85% of the study period and the maximum spatial cluster size to be ≤25% of the total population. Statistical significance of SaTScan-identified clusters were determined by Monte Carlo hypothesis testing employing 999 random replications under the null hypothesis to ensure adequate power for detecting clusters. A *P*-value less than 0.05 was considered to be statistically significant^36^,^37^.

We conducted spearman correlation analyses within each detected cluster to examine the associations between the monthly SFTS incidence and the climate variables at different time lags. For the spearman correlation analyses, first, climatic data (including monthly average temperature and relative humidity, and monthly cumulative rainfall and sunshine hours) covering 700 surveillance stations in mainland China during the study period were obtained from the China Meteorological Data Sharing Service System (http://cdc.cma.gov.cn). The data were spatial interpolated down to a 1 km^2^ resolution grid using Kriging model, and then the monthly average values for each cluster were calculated in ArcGIS 9.2. Second, the associations between the monthly SFTS incidence and the climatic variables at different time lags within each cluster were calculated by spearman correlation analyses, and the time lag associated with the maximum correlation coefficient was calculated as well, based on which parameter estimates with 95% confidence intervals (CIs) were reported^38^.

### Risk Assessment

A boosted regression tree (BRT) model coupled with the maximum entropy method was applied at the county level to assess the risk factors associated with the occurrence of human SFTSV infections in China. BRT modeling is efficient for predicting distributions of organisms while accounting for non-linear relationships and interactions between covariates, and it has been widely used for mapping the risk of infectious diseases^39^,^40^,^41^,^42^,^43^. In our BRT Model, all counties with SFTS cases were considered as positive counties, and five times as many negatives were selected randomly from all SFTS-absent counties. A bootstrapping procedure was employed to provide a robust estimation of model parameters. A tree complexity of 5, a learning rate of 0.005, and a bag fraction of 75% were used to identify the optimal number of trees. The relative contribution of each variable was estimated from the identified trees and served as an indicator of each variable's relative importance for predicting the risk of SFTS. For the bootstrapping procedure, the following sequential steps were repeated 50 times: first, 890 counties were randomly selected without replacement from all 2744 counties without SFTS cases throughout China, and were then combined with the 178 counties with SFTS cases to form a balanced bootstrap dataset (1-to-5 case-control ratio). Second, a training dataset with 75% of the points and a test dataset with 25% of the points were randomly selected from the current dataset. Third, a BRT model was built using the training set, and then the model was validated using the test set; receiver-operating characteristic (ROC) curves and area under the curve (AUC) were produced to estimate the predictive power of the model. Finally, a risk map of SFTSV human infections was created based on the average predicted probabilities overall bootstrap datasets. All statistical analyses were performed using R software (version 3.1.1; R Core Team 2014).

## Supplementary Material

Supplementary Information Supplementary Information

## Figures and Tables

**Table 1:** Epidemiologic features of human SFTS cases in China from 2010 to 2013

Characteristic	Total cases (n = 1768)	Deaths (n = 145)
Demographic feature		
Female, No. (%)	911 (51.5%)	69 (47.6%)
Age, median (range)	61 (1–93)	66 (40–86)
Age, mean ± SD	60.6 ± 12.2	65.2 ± 10.5
Famers/forest workers	1669 (94.4%)	138 (95.2%)
Time from onset to admission, median (range)	6 (0–220)	7 (0–146)
Temporal distribution feature		
2010	53	8
2011	461	46
2012	578	43
2013	676	48
Epidemic peak, No. (%)	May–July, 1188 (67.2%)	May–July, 98 (67.6%)
Spatial distribution feature		
No. provinces	14	7
No. counties	178	59
Severely affected Provinces (No.)		
1	Henan (688)	Shandong (58)
2	Shandong (504)	Hubei (32)
3	Hubei (219)	Henan (19)
4	Liaoning (120)	Liaoning (12)
5	Anhui (116)	Anhui (12)
6	Zhejiang (65)	Zhejiang (8)
7	Jiangsu (41)	Jiangsu (4)

**Table 2:** Information for spatial-temporal clusters of SFTS in China from 2010 to 2013

Clusters*	1	2	3
Time period	2011/4–2013/10	2010/5–2013/11	2011/5–2013/11
No.obs	752	311	159
No.exp	9	13	14
RR	152.9	30.0	12.4
LLR	2811.3	728.6	248.1
Annual incidence	3.00/100,000	0.90/100,000	0.40/100,000
No.counties	17	18	24
No.population	11,989,480	10,206,346	16,331,687
Area (Km^2^)	36183.7	23382.7	20476.5
Climate feature	humid subtropical climate	warm temperate humid monsoon climate	warm semi-humid continental monsoon climate
Major geomorphology	wooded and hilly area	mountainous and hilly area	low mountain-hill and plains
Elevation, median(range)	88 m (4–1538 m)	78 m (0–810 m)	193 m (1–1378 m)
Primary ticks	*H. longicornis*	*H. longicornis*	*H. longicornis*

Cluster* 1 belongs to primary cluster, 2 and 3 belong to secondary clusters.

No.obs: number of observed cases; No.exp: number of expected cases;

RR: relative risk for the SFTS incidence in the cluster compared to the national average incidence at the same time period; LLR: log likelihood ratio; No.counties: number of counties within cluster;

No.population: population within the cluster.

**Table 3:** Summary of the relative contributions (%) of predictor variables for the SFTS data in the boosted regression trees model

Variable	Boosted regression trees	
	Relative contribution (mean)	Relative contribution (sd)
Temperature*	12.44	1.72
Rainfall*	13.82	1.39
Relative humidity*	8.29	1.97
Sunshine hours*	13.36	2.14
Elevation*	19.15	2.31
Percentage coverage of forest*	5.16	1.22
Percentage coverage of shrub	2.57	1.04
Percentage coverage of cropland	3.85	1.18
Cattle density*	6.24	1.65
Goat density	2.43	0.73
Population density	2.52	1.24
Haemaphysalis longicornis^a^*	9.91	1.52
*Rhipicephalus microplus*^a^	0.26	0.20

*Variables whose relative contribution in the BRT models more than 5 were considered to be significantly contributes to the occurrence of human infection with SFTSV.

a*Haemaphysalis longicornis* and *Rhipicephalus microplus* are binary variables, whether the tick presence or absence in each county.
