# Supplementary material for: A National Assessment of the Epidemiology of Severe Fever with Thrombocytopenia Syndrome, China
Source: Sci Rep. 2015 Apr 23;5:9679. doi: 10.1038/srep09679 (PMC4407178; doi:10.1038/srep09679)
Supplement: Supplementary Information [file srep09679-s1.pdf]

Supplementary Information:

A National Assessment of the Epidemiology of Severe Fever with Thrombocytopenia Syndrome, China

Kun Liu[1†], Hang Zhou[2†], Ruo-Xi Sun[1,3], Hong-Wu Yao[1], Yu Li[2], Li-Ping Wang[2], Di Mu[2], Xin-Lou Li[1], Yang Yang[4], Gregory C. Gray[5], Ning Cui[6], Wen-Wu Yin[2], Li-Qun Fang[1]*, Hong-Jie Yu[2]*, and Wu-Chun Cao[1]*

[1] The State Key Laboratory of Pathogen and Biosecurity, Beijing Institute of Microbiology and Epidemiology, Beijing 100071, P. R. China.

[2] Division of Infectious Disease, Key Laboratory of Surveillance and Early-warning on Infectious Disease, Chinese Centre for Disease Control and Prevention, Beijing 102206, P. R. China.

[3] Anhui Medical University, Hefei, 230032, P. R. China.

[4] Department of Biostatistics, College of Public Health and Health Professions, and Emerging Pathogens Institute, University of Florida, 32311, Florida, USA.

[5] Duke University School of Medicine, Durham, 27710, North Carolina, USA.

[6] The 154 Hospital, People's Liberation Army, Xinyang, 464000, P.R. China.

1. **Table S1-S2**
2. **Figure S1-S6**
3. **Search strategy and selection criteria**

Table S1-S2

Table S1. Description of potential influencing factors used in the analyses.

Table S2. Spearman correlation coefficient (95% confidence intervals) between monthly incidence of SFTS and climatic variables.

Figure Legends

Figure S1. Age and sex distribution of the SFTS incidence and CFRs in Henan Province and all other provinces.

Figure S2. Seasonal epidemic curves of SFTS incidence in the severely affected provinces. The SFTS epidemic peaked later in provinces with higher latitudes. From south to north, the peak months were May to July in Henan, Anhui and Hubei provinces combined (most cases appeared in the junction region of the three provinces), between June and July in Shandong Province, and between July and August in Liaoning Province**.**

Figure S3. Relationships between risk factors and SFTS occurrence in the BRT model. The figure was created in R software (version 3.1.1; R Core Team 2014).

Figure S4. ROC curves of the predicted risk of SFTS occurrence in the BRT model. The grey lines are the ROC curve for each repeat, and the solid, dashed and dotted lines indicate the average ROC curve of 50 repeats based on the bootstrapping procedure for the train set, test set, and the prediction, respectively. The figure was created in R software (version 3.1.1; R Core Team 2014).

Figure S5. The typical ecological environment with tea-picking activities in Xinyang Prefecture of Henan Province, the region most highly endemic for STFS in China. The photographs were taken in Xinyang Prefecture by Ning Cui.

Figure S6. The geographic distribution of two tick species which have been associated with SFTS in China. The indicoliate green area represents the distribution of *Rhipicephalus microplus* ticks in China, and the yellow area represents the distribution of *Haemaphysalis longicornis* ticks in China. The map was created in ArcGIS 9.2 software (ESRI Inc., Redlands, CA, USA).

Search strategy and selection criteria:

We searched PubMed and ISI Web of Science for literatures published in English, and WanFang database, China National Knowledge Infrastructure, and Chinese Scientific Journal Database for literatures published in Chinese till the date of December 31, 2013. Search terms included were "*Haemaphysalis longicornis*", or "*Rhipicephalus microplus*", in combination with "China". We also researched books and surveys from the Chinese Ministry of Health, Chinese Center for Diseases Control and Prevention, institutes, and universities in China in the past 30 years. Then, we extracted the distribution of ticks at the prefecture or county level from the literatures.

Table S1. Description of potential influencing factors used in the analyses.

Variables	Description (Unit)	Type
Temperature	Average temperature during the study period for each county (℃)	Continuous
Rainfall	Accumulative rainfall during the study period for each county (mm)	Continuous
Relative humidity	Average relative humidity during the study period for each county (%)	Continuous
Sunshine hours	Accumulative sunshine hours during the study period for each county (hours)	Continuous
Elevation	Average elevation for each county (m)	Continuous
Percentage coverage of forest	Percentage coverage of forest for each county (%)	Continuous
Percentage coverage of shrub	Percentage coverage of shrub for each county (%)	Continuous
Percentage coverage of cropland	Percentage coverage of cropland for each county (%)	Continuous
Goat density	Goat density for each county (head per km^2)	Continuous
Cattle density	Cattle density for each county (head per km^2)	Continuous
Population density	Population density for each county (1000 persons per county)	Continuous
Haemaphysalis longicornis	Presence or absence in each county	Categorical
Rhipicephalus microplus	Presence or absence in each county	Categorical

Table S2. Spearman correlation coefficient (95% confidence intervals) between monthly incidence of SFTS and climatic variables.

Cluster	Temperature	Rainfall	Relative humidity	Sunshine hours
Cluster 1	L_0=0.76	L_0=0.60	NS	L_0=0.66
	(0.51 to 0.88)	(0.34 to 0.78)		(0.37 to 0.84)
Cluster 2	L_0=0.86	L_0=0.59	L_0=0.62	L_2=0.41
	(0.75 to 0.91)	(0.34 to 0.75)	(0.36 to 0.79)	(0.08 to 0.67)
Cluster 3	L_0=0.92	L_0=0.59	L_0=0.46	L_2=0.62
	(0.84 to 0.94)	(0.27 to 0.81)	(0.12 to 0.69)	(0.32 to 0.81)

L_x: the lagged months. NS: not significant.

Figure S1

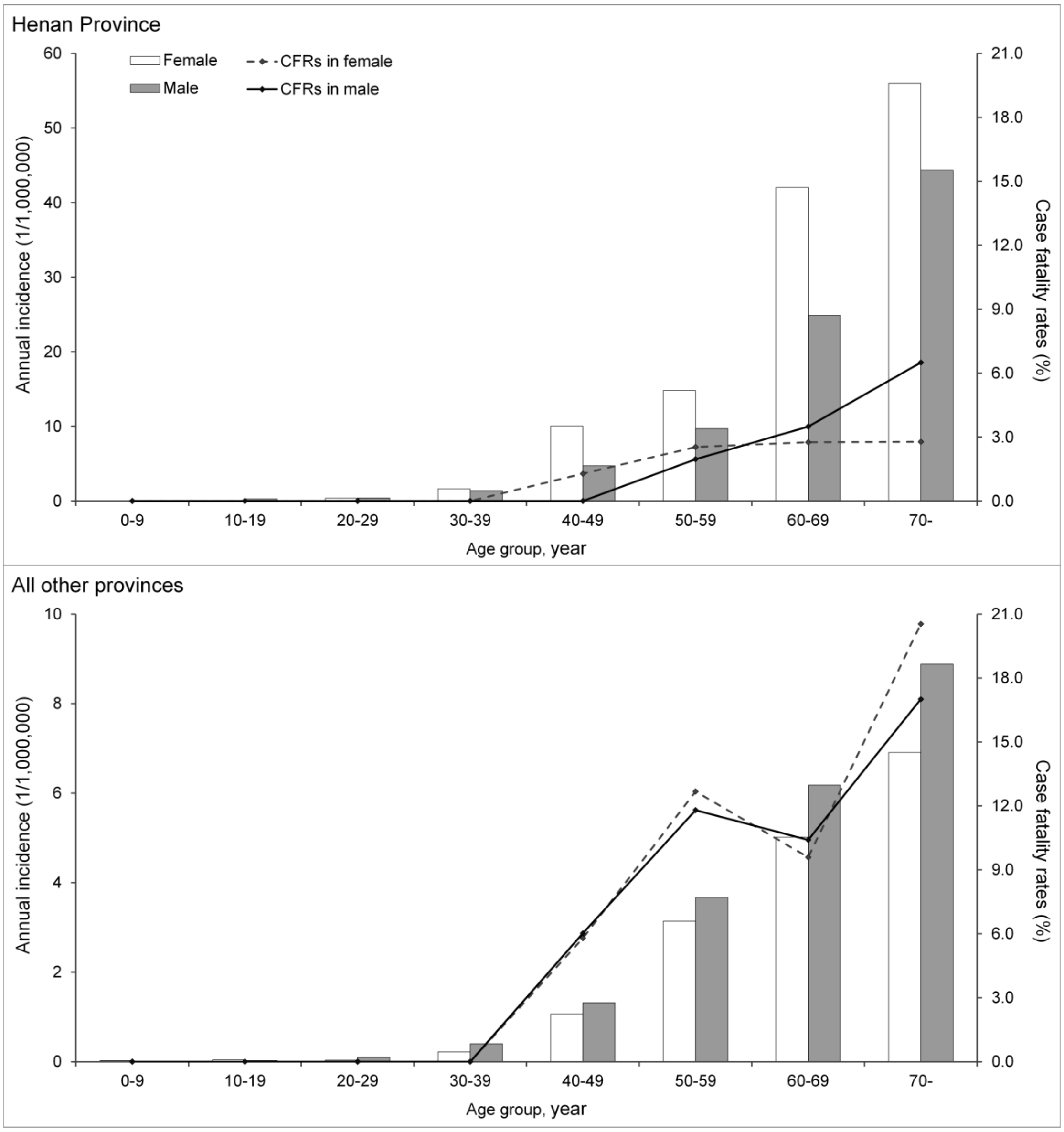

Henan Province
Female
Male
CFRs in female
CFRs in male
Annual incidence (1/1,000,000)
Case fatality rates (%)
60
50
40
30
20
10
0
21.0
18.0
15.0
12.0
9.0
6.0
3.0
0.0
0-9
10-19
20-29
30-39
40-49
50-59
60-69
70-
Age group, year
All other provinces
Annual incidence (1/1,000,000)
Case fatality rates (%)
10
8
6
4
2
0
21.0
18.0
15.0
12.0
9.0
6.0
3.0
0.0
0-9
10-19
20-29
30-39
40-49
50-59
60-69
70-
Age group, year

Figure S2

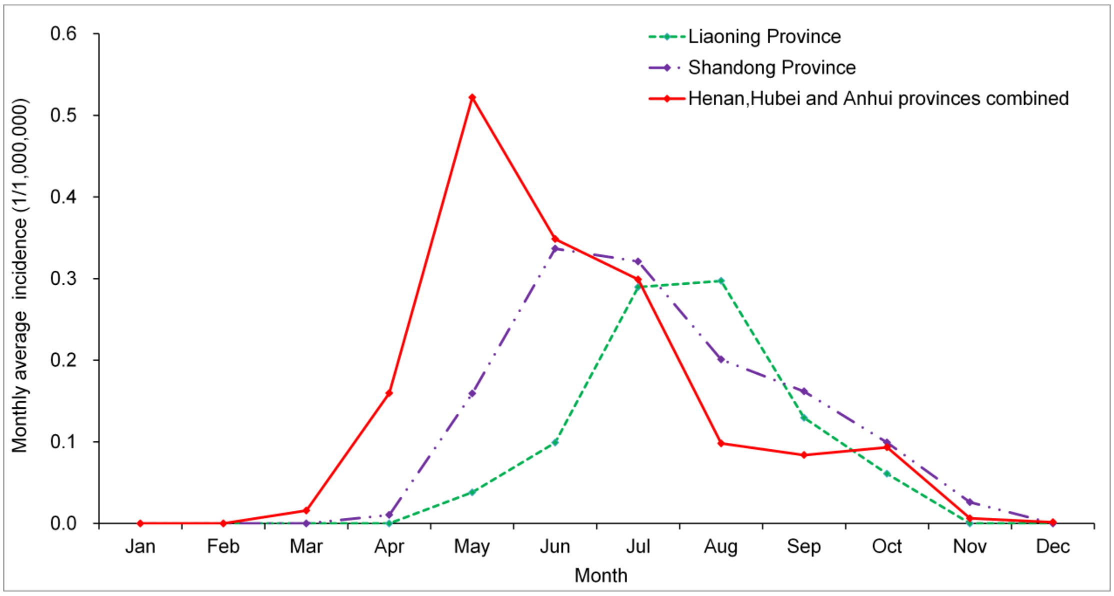

Liaoning Province
Shandong Province
Henan,Hubei and Anhui provinces combined
Monthly average incidence (1/1,000,000)
0.6
0.5
0.4
0.3
0.2
0.1
0.0
Jan
Feb
Mar
Apr
May
Jun
Jul
Aug
Sep
Oct
Nov
Dec
Month

Figure S3

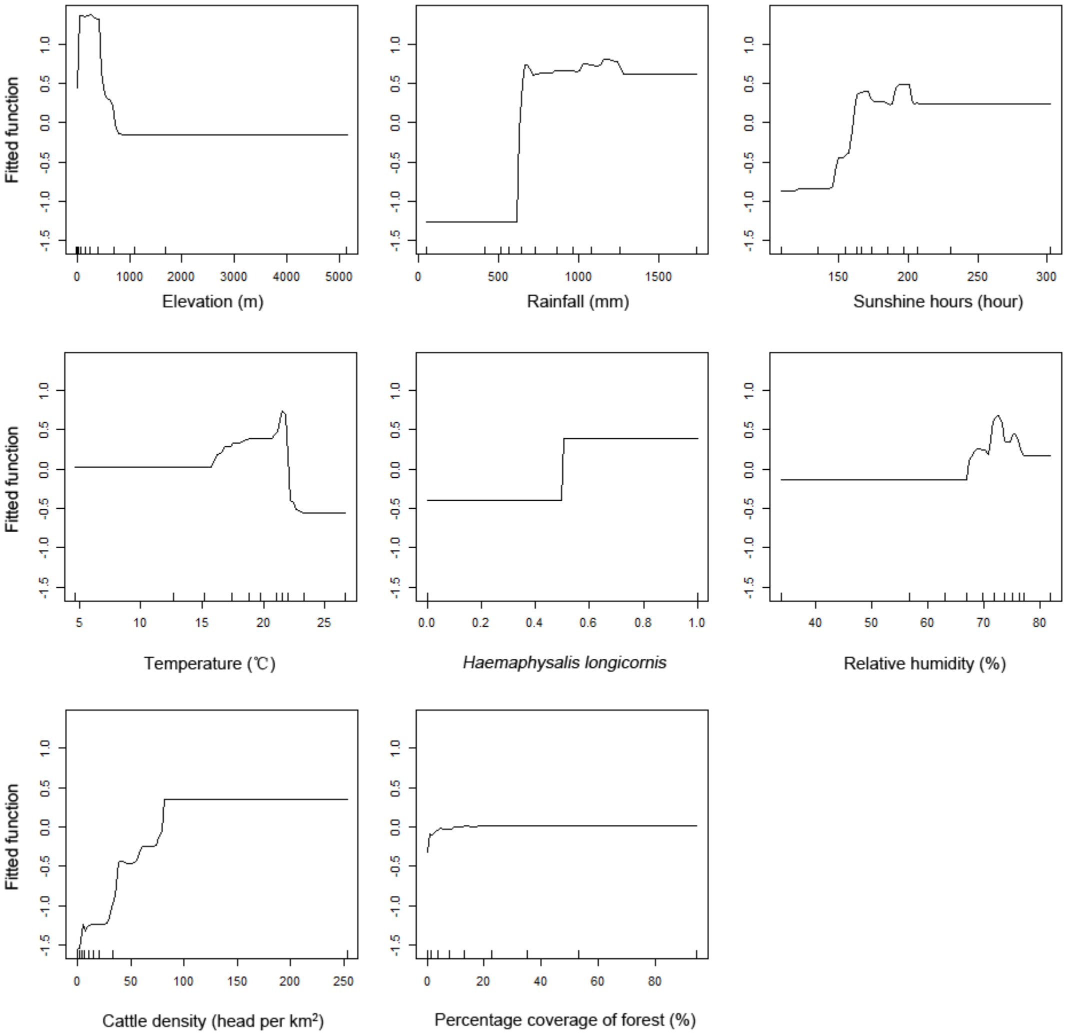

Fitted function
Elevation (m)
Rainfall (mm)
Sunshine hours (hour)
Temperature (℃)
Haemaphysalis longicornis
Relative humidity (%)
Cattle density (head per km²)
Percentage coverage of forest (%)

Figure S4

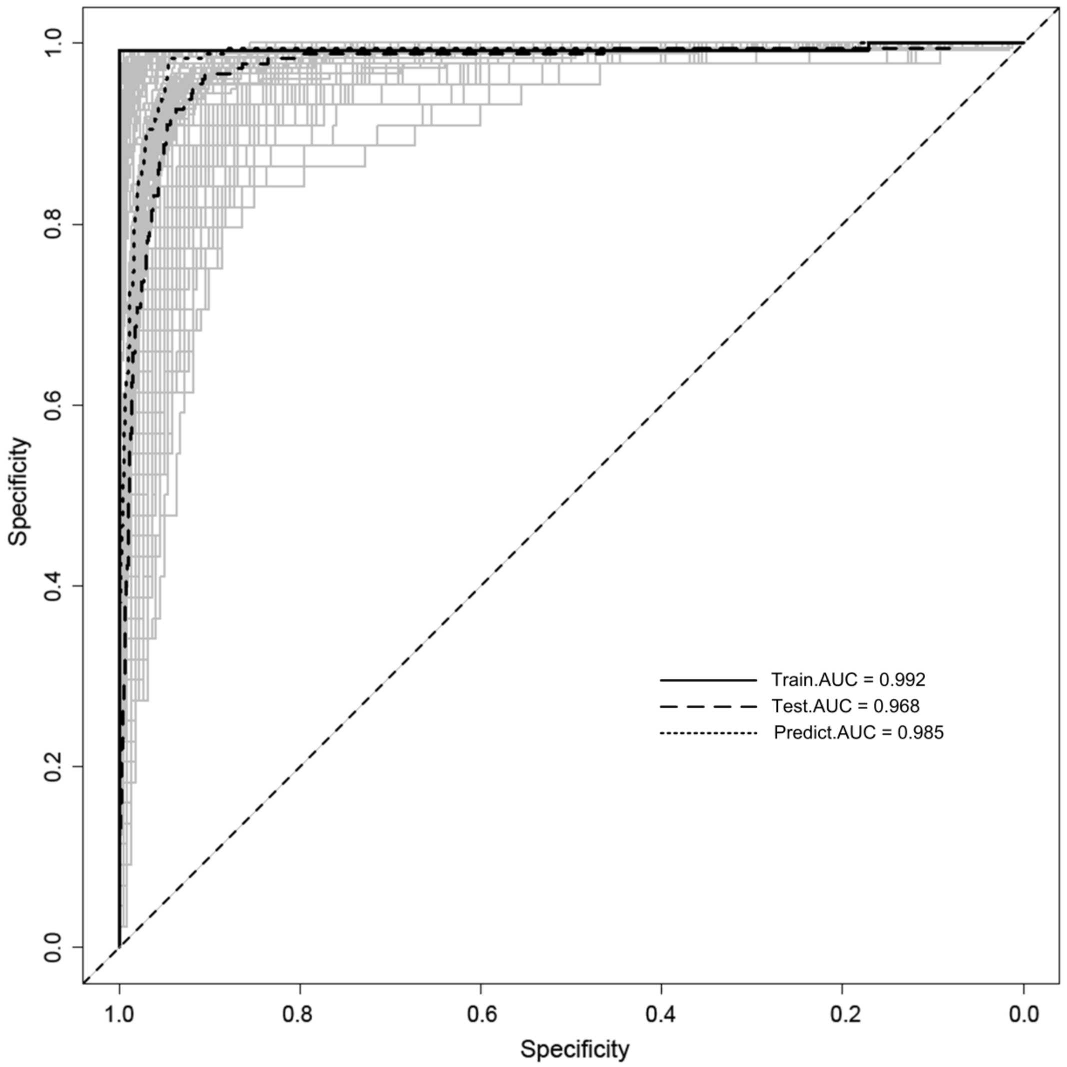

Specificity
1.0
0.8
0.6
0.4
0.2
0.0
Train.AUC = 0.992
Test.AUC = 0.968
Predict.AUC = 0.985

Figure S5

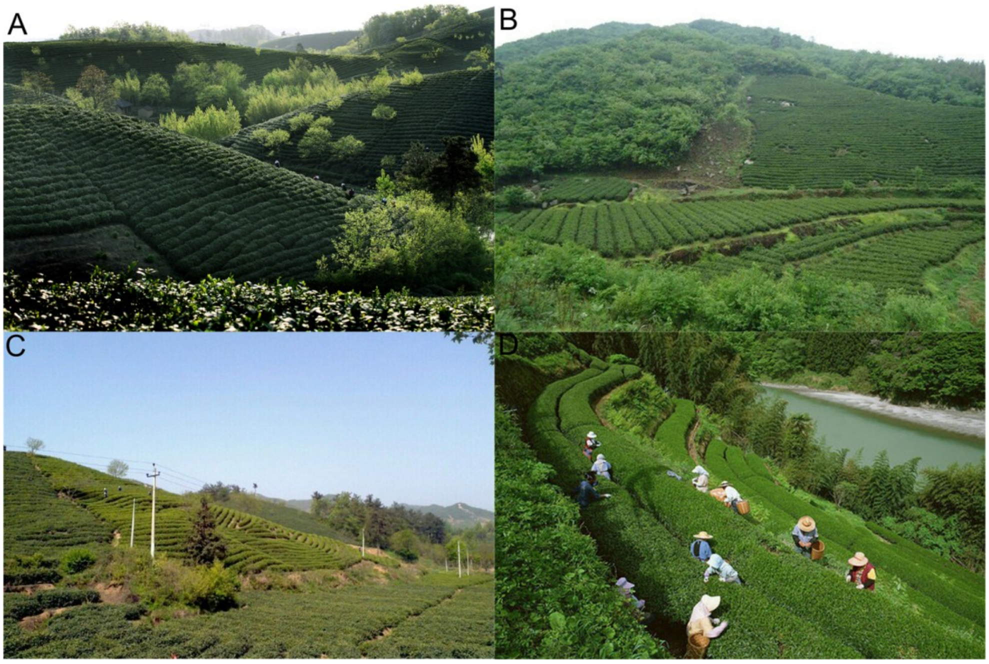

Figure S6

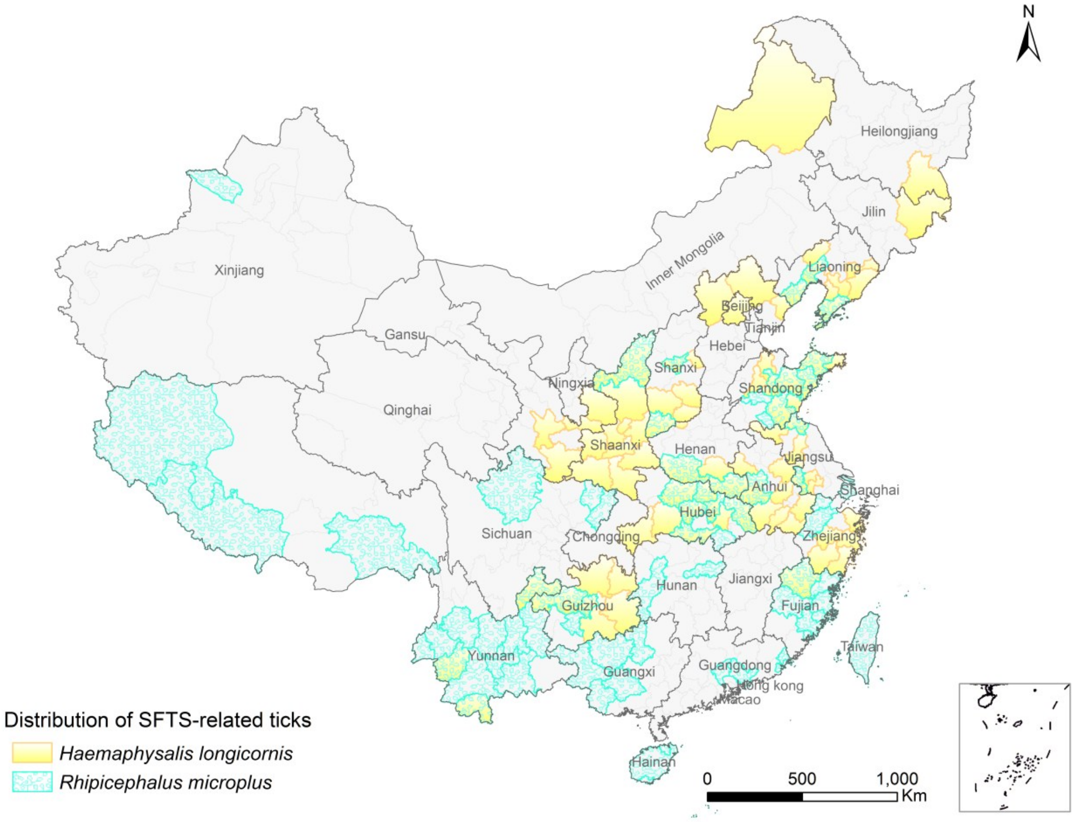
N
Heilongjiang
Jilin
Liaoning
Inner Mongolia
Xinjiang
Gansu
Beijing
Tianjin
Hebei
Ningxia
Shanxi
Shandong
Qinghai
Shaanxi
Henan
Jiangsu
Anhui
Shanghai
Hubei
Sichuan
Chongqing
Zhejiang
Jiangxi
Hunan
Guizhou
Fujian
Taiwan
Yunnan
Guangxi
Guangdong
Hong kong
Macao
Hainan
Distribution of SFTS-related ticks
Haemaphysalis longicornis
Rhipicephalus microplus
0
500
1,000
Km
